# Surgical treatments on adult tethered cord syndrome: A retrospective study

**DOI:** 10.1097/MD.0000000000005454

**Published:** 2016-11-18

**Authors:** Jun Gao, Xiangyi Kong, Zhimin Li, Tianyu Wang, Yongning Li

**Affiliations:** a Department of Neurosurgery, Peking Union Medical College Hospital, Chinese Academy of Medical Sciences, Beijing, People's Republic of China; b Department of Anesthesia, Critical Care and Pain Medicine, Massachusetts General Hospital, Harvard Medical School, Harvard University, Boston, MA.

**Keywords:** microsurgery, tethered cord syndrome, tumor

## Abstract

To investigate effects of surgical treatment on adult tethered cord syndrome (TCS).

A retrospective analysis of 82 adult patients (17 male cases, 82% and 24 female cases, 59%) with TCS treated by surgery was conducted between March, 2005 and December, 2015, with an average age of 31.6 years and average disease course of 6.7 years. All the 82 cases of patients received nerve electrophysiology monitoring assisted microsurgery. After surgery, all patients were followed up for an average of 2.5 years. Surgical effects were evaluated according to Hoffman grading system. As this is just a retrospective study that does not involve any interventions, ethical approval was not necessary according to the rules of the hospital.

All patients were followed up, no death occurred. According to Hoffman grading system, the neurologic symptoms were improved in 22 patients (27%), stabilized in 60 patients (73%). Of 10 cases with lipoma tethered spinal cord, corresponding symptoms were improved in 2 cases. Of 32 cases with tethered spinal cord caused by dermoid cyst and epidermoid cyst, the symptoms were improved in 6 cases. Of 40 cases without occupying lesions of tethered spinal cord, the symptoms were improved in 14 cases. Besides, there was no deteriorated case.

Surgical treatment on adult patients with TCS can improve the neurologic deficits which are associated with the course of disease, early treatment has much better curative effect.

## 1 Introduction

Tethered cord syndrome (TCS) refers to a series of neurological dysfunction caused by the retraction of the conus medullaris related to a variety of reasons, which is manifested by both lower extremities orbicularis weakness, sensory abnormalities, defecation dysfunction, and so on.^[1]^. In the early stage of embryo, spinal cord and vertebral canal were roughly equal, but in the later development process, bony spinal growth were indicated to be faster, which was out of synchronization with the growth of spinal cord.^[2]^ As for normal embryo under 20 weeks, the termination of spinal cord was located at the level of L4 to L5, and at the level of L3 under 40 weeks, when the baby was born, it was located at the level of L1 to L2.^[3]^ Deformity of spinal cord, local tumor compression, scar adhesion, stubby filum terminale, can cause spinal cord fixed to the lesion site, so that the spinal cord cannot move up normally, which is the basis contributing to the incidence of TCS.^[3,4]^ Adult onset cases are rare compared to that in children.^[2]^ The tumor compression of the cone and the tail is one of the main causes for the tethered cord.^[2]^

In 1886, Von Reeklinghausen reported autopsy results of the patients with lumbosacral hypertrichosis accompanied with spina bifida, showing that the spinal cord was adhered to fat in the lumbosacral region, conus terminalis was indicated to be tensed.^[4]^ In 1953, Garceau described the “filum terminal syndrome,” suspected that the tensive filum terminal pulled the spinal cord might cause defecation dysfunction and other symptoms.^[5]^ In 1976, Hoffman et al^[6]^ reported 31 patients combined with conus medullaris after stretching slenderization, corresponding nerve function were improved following cutting off the tensive and thicker and filum terminale; besides, syndrome that the conus medullaris was stretched was named “tethered spinal cord syndrome,” has been used to describe for nervous dysfunction caused by conus medullaris stretching.^[6]^ In 1981, Yamada et al^[7]^ found in animal studies that the role of mitochondrial metabolism was reduced in the termination of spinal cord, the greater the tension, the longer the time, and the more serious the nervous dysfunction was. Therefore, he believed that the dysfunction of the nerve cells in the spinal cord might be caused by the impairment of mitochondrial oxidative metabolism.^[7]^ Significantly different from children with TCS, there was also the existence of incentives in adult patients with TCS, such as hip hyperflexion due to high falling injury, lithotomy position delivery, long-time sitting causing over stretching of the spinal cord, all of which might evoke the development of TCS.^[8]^

To investigate effects of surgical treatment on adult TCS, a retrospective analysis of 82 adult patients with TCS treated by surgery was conducted between March 2005 and December 2015.

## 2 Materials and methods

### 2.1 Subjects

A retrospective analysis of 82 adult patients with TCS treated by surgery was conducted between March 2005 and December 2015 in Peking Union Medical College Hospital. All the patients were from China and of Asian ethnicity. Of these patients, there were 34 males and 48 females, with an age range of 18 to 47 years (average age, 31.6 years), and average disease course of 6.7 years. There were 10 cases of lumbosacral intraspinal canal lipoma (12%), 32 cases of (39%) dermoid cyst and epidermoid cyst, and 40 cases (49%) without occupying lesions of tethered spinal cord. In addition and preoperatively, there were 68 cases (83%) of varying degrees of pain in the lumbosacral portion and lower extremity, 58 cases (71%) of motor dysfunction of the lower extremity, 44 cases (54%) with abnormal sensation, and 50 cases (61%) of defecation dysfunction.

### 2.2 Imaging examination

**Figure 1:**
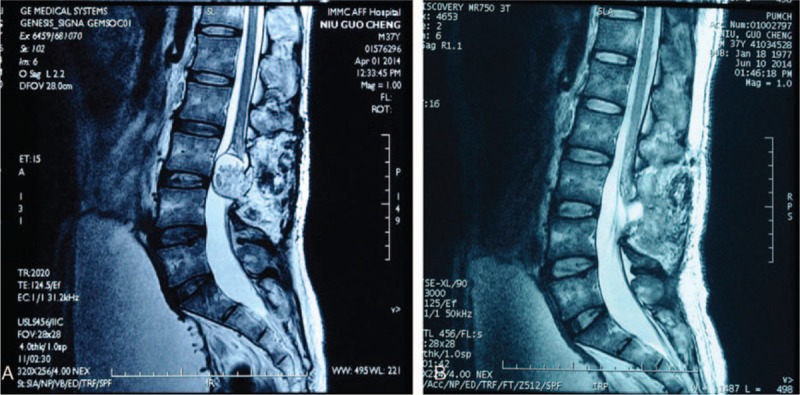
(A) A 37-year-old male patient with a lumbar spinal lipoma at L3/L4 level. He presented with symptoms of lower back pain and legs pain. After surgery, the lipoma was removed almost completely (B).

All of the included 82 cases of patients received preoperative enhanced magnetic resonance imaging (MRI) examination, and there were several characteristics listed as follows: thickened filum terminale in a diameter of >2 mm; elongated, tapering, and low position of the coni medullaris, the coni medullaris located below the plane of vertebral body (L2) was considered to be low position of the coni medullaris; coni medullaris or the filum terminale attached closely to the posterior wall of the thecal sac, in a relatively straight shape; a large subarachnoid space was existed in the sacral canal; possible existence of occupying lesions adhered to the coni medullaris or the cauda equina, such as lipoma, dermoid cyst, and epidermoid cyst; potential existence of myelomeningocele or changes after prosthesis (Fig. 1A and B).

### 2.3 Surgical procedures

All patients received general anesthesia and took their prone position, neural electrophysiological monitoring electrode were then placed, followed by the acquisition and collection of muscle electromyography signals from the anal sphincter, bilateral musculus vastus lateralis, gastrocnemius and mesothenar. A total of 72 cases applied positive straight incision, 10 cases of lumbosacral lipoma with longitudinal incision. After exposing the dura mater spinalis, it was cut from the normal anatomical structure to the lesion. Cauda equina was managed by sharp releasing adhesion under the nerve electrophysiological monitoring, tumors were removed with the use of medical ultrasonic dissector. After the tumor was removed, the dura mater spinalis with low tonus was closed by water, and the dura mater spinalis with high tonus was formed by the autogenous fascia. For patients combined with subcutaneous giant lipoma in the lumbosacral region, the subcutaneous tumor was removed, and the drainage tube was placed into the left empty cavity, followed by pressurized dressing and vacuum aspiration.

### 2.4 Follow-up

All patients received a 0.5 to 3.5-year follow-up by outpatient or telephone, with an average follow-up period of 2.5 years. Analysis was performed according to Hoffman grading system. There were 4 cases of patients with grade 0 by preoperative Hoffman grading, 20 cases with grade 1, 28 cases with grade 3, 18 cases with grade 4, 10 cases with grade 5, and remaining 2 cases with grade 6. Surgical effects were evaluated by observing improvement of symptoms of each patient postoperatively.

## 3 Results

All patients were followed up, no death occurred. Changes of symptoms were associated with the course of disease; patients with relatively shorter disease course were shown to have a mild Hoffman grading, whereas patients with relatively longer disease course were indicated to have a severe Hoffman grading. Meanwhile, patients with shorter disease courses were suggested to accompany with obvious improvement of symptoms postoperatively when compared to those patients preoperatively; besides, the course of disease was within 1 year regarding those patients showing a completely recovery of the abovementioned symptoms. According to Hoffman grading system, the neurologic symptoms were improved in 22 patients (27%) and stabilized in 60 patients (73%). By preoperative Hoffman grading, in the 20 cases with grade 1, 8 cases of patients indicated symptoms improvement to grade 0; in cases with grade 2, 6 cases of patients were improved and transferred to grade 1, and 2 cases of patients transferred into grade 0; in cases with grade 3, 4 cases of patients showed improved symptoms and changed into grade 2; in cases with grade 4, 2 cases were improved and changed into grade 3. Among them, lipoma-oriented TCS was found in 10 cases of patients, of which including 2 cases showing symptoms improvement, 8 cases showing symptom stabilization, no case got worse. Dermoid cyst and epidermoid cyst caused TCS was observed in 32 cases, with 6 cases indicating improved symptoms, 26 cases showing stabilized symptoms, and no case got worse. Of 40 cases without occupying lesions of TCS, the symptoms were improved in 14 cases and stabilized in 26 cases, there was no deteriorated case. The surgical release degrees for TCS with different pathologic changes are shown in Table 1. The operation curative effects with curative rates for TCS with different symptoms, and signs are shown in Table 2 in detail.

**Table 1:**
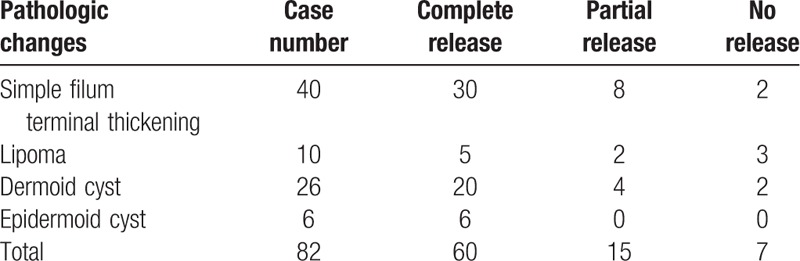
Surgical release degrees for TCS.

**Table 2:**
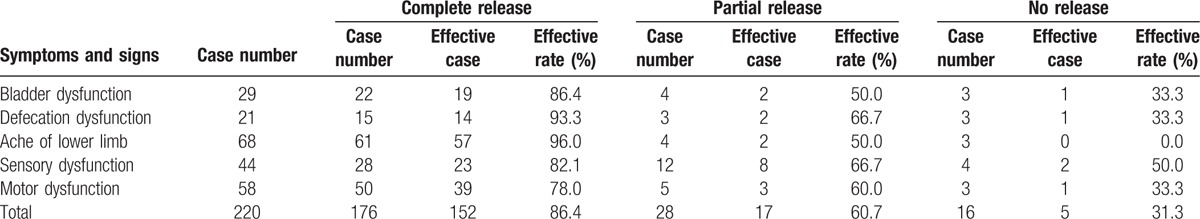
The operation curative effects for TCS with different symptoms.

Following postoperative lumbar spine reexamination by MRI, corresponding results were as follows: postoperative position of coni medullaris was relatively improved than that of the preoperative, and the width was broadened to the posterior wall of the thecal sac; the tension of filum terminale was decreased, filum terminale, and cauda equina showed relatively normal morphology, filum terminale apart from the posterior wall of the thecal sac was found in a part of cases of TCS patients; and if there was occupying lesions, volumes of occupying lesions showed decreased trends and indicated alleviated adhesion or compression to the coni medullaris or cauda equina. Figure 1A shows a 37-year-old male patient with a lumbar spinal lipoma at L3/L4 level. He presented with symptoms of lower back pain and legs pain. After surgery, the lipoma was removed almost completely (Fig. 1B). The patient was followed up for 2 years without local recurrence.

As for the postoperative complications, there were 4 cases (5%) of spinal fluid leakage, and the 2 patients were cured following vacuum aspiration and pressurized dressing; there were 6 cases (7%) showing delayed wound healing, mainly caused by spinal fluid leakage or fat liquefaction. Besides, there was no case of infection, new onset of nerve injury or second TCS postoperatively.

## 4 Discussion

Surgical treatment is the only effective method to relieve occupying, loose adhesions, and compression, its main purpose is to lift the tethered to reduce the stretching of the taper tension, and thus to control further development of symptoms and to reduce further damage to the nerve function. Through the follow-up of 56 cases of adult TCS patients, Hüttmann et al^[9]^ found that the pain relief rate was 86%, which was the most obvious symptoms that alleviated, remission rate of the lower limb spasticity was 7l%, and the remission rate of bladder dysfunction and feeling movement dysfunction was 44% and 35%, respectively. In this group of patients, postoperative pain symptoms of lumbosacral portion and both lower extremities improved significantly and remarkably, the defecation dysfunction in most patients was improved to some extent, but there were still some patients having frequent micturition and urinary retention; furthermore, muscle strength of lower limbs also increased, most patients had different degrees of improvement of muscle strength, which was basic consistent with the conclusion draw from Htittmann. Through the long-term follow-up, patients with a shorter duration, lighter TCS degree, generally the prognosis would be good, and symptoms improved significantly; on the other hand, for patients with longer course of disease, serious TCS, and higher frequency that tumor wrapped around the cauda equina, corresponding surgery effect was not so obvious; some patients even showed no improvement of symptoms, and the risk of postoperative TCS was relatively high.^[10]^ Of course, if the relief of tethered parts of the cauda equina obtained a relatively satisfactory outcome during the surgery, most occupying lesions and diseased filum terminale were removed, postoperative symptoms improved at different degrees, further recovery of the nerve function could thus be observed in the long-term follow-up period. It is recommended that routine examination of filum terminale should be performed in the operation, associated with the disconnection of the diseased filum terminale subject to adhesion or thickening and shortening.^[11]^ In this paper, we suggest that it is possible to cut off the filum terminale if there will have no injury to the nerve under electrophysiological monitoring, corresponding prognostic outcome will be better than that that without disconnection of filum terminale.

TCS caused by different causes may have different curative effects following surgical treatment, for example, if TCS is induced by simply thickening filum terminale, the removal of filum terminale can get better operation results; if it is caused by myelomeningocele, which are usually combined with spina bifida, the operation is relatively complex and surgery is needed to be operated as soon as possible to protect the neurological function, the most important is to suture the dura completely and prevent further TCS.^[2]^

Dermoid cyst should be completely removed together with cyst wall as much as possible, cystic wall residue is easy to cause the recurrence of cyst, although decreased volume of cyst can relieve the symptom of tethered, characteristics of dermoid cyst may lead greater possibility to TCS than lipoma. For larger cysts, it is not possible to force the free capsule wall directly, because the cone and the cauda equina are in a high tension state, and any tiny stretching is likely to cause further damage. Therefore, it is necessary to remove contents within the cyst and to reduce the size of the cyst as far as possible, followed by free cystic wall, and then to minimize the stretching of the nerve tissue. For cyst wall with many serious adhesions of cauda equina nerve, partial resection of the cyst wall can be performed under electrophysiological monitoring, which will also have a good operation effect. But previous investigation estimated that no more than 40% of dermoid cyst could be completely removed.^[12]^

The possibility of self-growth of lipoma is relatively low, and it is closely related to the increase or decrease of fats from other parts of the body.^[13]^ The growth of body weight and the use of hormones may cause the increase of lipoma and increased symptoms of TCS. The low growth ability of lipoma also leads to the problem that whether the tumor should be removed completely or not. Klekamp^[14]^ advocated that for small lipoma and cone did not show obvious compression, the symptom is mainly caused by tethered, simply releasing of the tethered is suitable to prevent postoperative adhesion and not to destroy lipoma; and for larger lipoma compressed the conus medullaris, decreasing the volume of lipoma from internal, retaining the capsule, and sewing up the incision will be more effective to reduce the possibility of adhesion. At present, the classification of lipoma-oriented TCS is confused, Arai et al^[15]^ had classified it into 5 different kinds, including the dorsal, caudal, combined, filar, and lipomyelomeningocele; while it was subdivided into the lower conical, lateral conical, and upper conical by Wang et al.^[16]^ On the whole, patients with filar TCS had the lightest symptoms, corresponding surgery was relatively easy, and prognosis in the follow-up period was relatively better after removing filum terminale. Symptoms in patients with combined and lipomyelomeningocele TCS was relatively heavier, fat surrounded multiple bundle of cauda equina, dissociating of the cauda equina was therefore more difficult, and it was difficult to be completely removed, also accompanied with subsidiary-injury recurrence of TCS, finally resulting in poor prognosis and none significant improvement of symptoms.^[17]^

Yamada and Lonse^[18]^ divided 70 cases of adult TCS patients into 2 groups, who underwent surgical treatment and followed by comparative analysis, patients with longer course of disease were found to show limited relief of motor sensory dysfunction and bladder dysfunction; pain in the lumbosacral portion and both lower extremities was relieved 3 months after surgical relaxation of the tethered cords; and in patients with shorter disease duration following surgical lysis, motor sensory dysfunction and bladder dysfunction were improved significantly, pain symptoms also alleviated rapidly. By clinical analysis of 611 cases of patients with lipoma-oriented TCS, Cui et al^[19]^ proved that patients with no symptoms and mild symptoms obtained satisfactory postoperative curative effects, according to Hoffman grading evaluation of preoperative and postoperative changes of symptoms, whereas the curative effect was relatively poor in patients showing severe symptoms after operation, early surgical treatment was therefore recommended to obtain better curative effect. In our study, in patients with severe Hoffman grading and without satisfied remission of symptoms, there were tendencies of longer medical history, more complications, and complicated symptoms; and for patients with relatively short medical history, Hoffman grading was shown to be mild and postoperative symptoms were improved obviously, which were similar with the above conclusions. In addition, some patients refused to take surgical treatment, and their symptoms were further aggravated or new symptoms appeared followed by telephone or outpatient follow-up.^[20]^ Therefore, early diagnosis and early surgical treatment will be possible to obtain a better prognosis for patients with symptomatic adult TCS.

## Acknowledgments

We would like to thank our colleagues from the Department of Neurosurgery, Peking Union Medical College Hospital, Chinese Academy of Medical Sciences and Peking Union Medical College, and the Department of Anesthesia, Critical Care and Pain Medicine, Massachusetts General Hospital, Harvard Medical School, Harvard University.

## References

[1] Maurya VP Rajappa M Wadwekar V Tethered cord syndrome—a study of the short-term effects of surgical detethering on markers of neuronal injury and electrophysiologic parameters. *World Neurosurg* 2016; 94:239–247. 27422680 10.1016/j.wneu.2016.07.005

[2] Lew SM Kothbauer KF Tethered cord syndrome: an updated review. *Pediatr Neurosurg* 2007; 43:236–248. 17409793 10.1159/000098836

[3] Hertzler DA 2nd DePowell JJ Stevenson CB Tethered cord syndrome: a review of the literature from embryology to adult presentation. *Neurosurg Focus* 2010; 29:E1. 10.3171/2010.3.FOCUS1079 20593997

[4] Garg K Tandon V Kumar R Management of adult tethered cord syndrome: our experience and review of literature. *Neurol India* 2014; 62:137–143. 24823721 10.4103/0028-3886.132329

[5] Garceau GJ The filum terminale syndrome (the cord-traction syndrome). *J Bone Joint Surg Am* 1953; 35-A:711–716. 13069561

[6] Hoffman HJ Hendrick EB Humphreys RP The tethered spinal cord: its protean manifestations, diagnosis and surgical correction. *Childs Brain* 1976; 2:145–155. 786565 10.1159/000119610

[7] Yamada S Zinke DE Sanders D Pathophysiology of “tethered cord syndrome”. *J Neurosurg* 1981; 54:494–503. 6259301 10.3171/jns.1981.54.4.0494

[8] Stetler WR Jr Park P Sullivan S Pathophysiology of adult tethered cord syndrome: review of the literature. *Neurosurg Focus* 2010; 29:E2. 10.3171/2010.3.FOCUS1080 20594000

[9] Hüttmann S Krauss J Collmann H Surgical management of tethered spinal cord in adults: report of 54 cases. *J Neurosurg* 2001; 95:173–178. 11599833 10.3171/spi.2001.95.2.0173

[10] Liu JJ Guan Z Gao Z Complications after spinal anesthesia in adult tethered cord syndrome. *Medicine* 2016; 95:e4289. 27442670 10.1097/MD.0000000000004289 PMC5265787

[11] Selcuki M Mete M Barutcuoglu M Tethered cord syndrome in adults: experience of 56 patients. *Turk Neurosurg* 2015; 25:922–929. 26617143 10.5137/1019-5149.JTN.11700-14.1

[12] Bristow RG Laperriere NJ Tator C Post-operative radiotherapy for recurrent dermoid cysts of the spine: a report of 3 cases. *J Neurooncol* 1997; 33:251–256. 9195496 10.1023/a:1005739606895

[13] Horrion J Houbart MA Georgiopoulos A Adult intradural lipoma with tethered spinal cord syndrome. *JBR-BTR* 2014; 97:121. 25073248 10.5334/jbr-btr.43

[14] Klekamp J Tethered cord syndrome in adults. *J Neurosurg Spine* 2011; 15:258–270. 21599446 10.3171/2011.4.SPINE10504

[15] Arai H Sato K Okuda O Surgical experience of 120 patients with lumbosacral lipomas. *Acta Neurochir (Wien)* 2001; 143:857–864. 11685617 10.1007/s007010170015

[16] Wang XG Zhou YD Ji SJ Pathology and treatment of tethered cord syndrome with lipoma. *Chin J Pediatr Surg* 1995; 16:197–199.

[17] Murata Y Kanaya K Wada H Reduction of caudal traction force using dural sac opening rather than spinal cord detethering for tethered cord syndrome caused by lipomyelomeningocele: a case report. *Spine J* 2014; 14:e1–e3. 10.1016/j.spinee.2014.02.031 24613376

[18] Yamada S Lonser RR Adult tethered cord syndrome. *J Spinal Disord* 2000; 13:319–323. 10941891 10.1097/00002517-200008000-00008

[19] Cui ZG Xiu B Xiao K Application of microsurgical technique for intraspinal lipoma tethered cord syndrome: report of 611 cases. *Chin J Neurosurg* 2011; 27:1128–1131.

[20] Tyagi R Kloepping C Shah S Spinal cord stimulation for recurrent tethered cord syndrome in a pediatric patient: case report. *J Neurosurg Pediatr* 2016; 18:105–110. 26942269 10.3171/2015.12.PEDS14645

