# Surgical treatments on adult tethered cord syndrome: A retrospective study: Erratum

**DOI:** 10.1097/MD.0000000000007808

**Published:** 2017-08-11

In the article, “Surgical treatments on adult tethered cord syndrome: A retrospective study”,^[1]^ which appeared in Volume 95, Issue 46 of *Medicine*, a sentence in the abstract, “A retrospective analysis of 82 adult patients (17 male cases, 82% and 24 female cases, 59%)…” appeared incorrectly and should have appeared as “A retrospective analysis of 82 adult patients (34 male cases, 41.5% and 48 female cases, 58.5%)…” In Table 1, the totals in the Complete release and Partial release columns appeared incorrectly and should have appeared as seen in the table below.

**Table 1:**
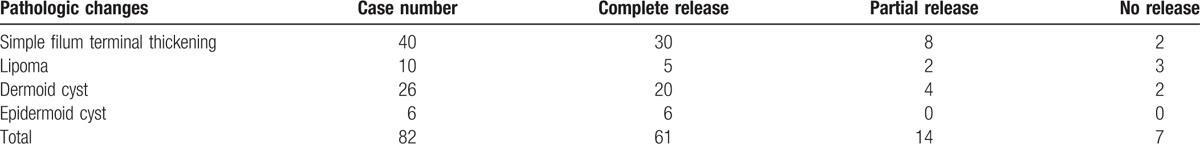
Surgical release degrees for tethered cord syndrome.

## References

[1] Gao J Kong X Li Z Surgical treatments on adult tethered cord syndrome: A retrospective study. *Medicine*. 2016 95:e5454. 27861396 10.1097/MD.0000000000005454 PMC5120953

